# DNA Binding of Centromere Protein C (CENPC) Is Stabilized by Single-Stranded RNA

**DOI:** 10.1371/journal.pgen.1000835

**Published:** 2010-02-05

**Authors:** Yaqing Du, Christopher N. Topp, R. Kelly Dawe

**Affiliations:** 1 Department of Plant Biology, University of Georgia, Athens, Georgia, United States of America; 2 Department of Genetics, University of Georgia, Athens, Georgia, United States of America; The University of North Carolina at Chapel Hill, United States of America

## Abstract

Centromeres are the attachment points between the genome and the cytoskeleton: centromeres bind to kinetochores, which in turn bind to spindles and move chromosomes. Paradoxically, the DNA sequence of centromeres has little or no role in perpetuating kinetochores. As such they are striking examples of genetic information being transmitted in a manner that is independent of DNA sequence (epigenetically). It has been found that RNA transcribed from centromeres remains bound within the kinetochore region, and this local population of RNA is thought to be part of the epigenetic marking system. Here we carried out a genetic and biochemical study of maize CENPC, a key inner kinetochore protein. We show that DNA binding is conferred by a localized region 122 amino acids long, and that the DNA-binding reaction is exquisitely sensitive to single-stranded RNA. Long, single-stranded nucleic acids strongly promote the binding of CENPC to DNA, and the types of RNAs that stabilize DNA binding match in size and character the RNAs present on kinetochores *in vivo*. Removal or replacement of the binding module with HIV integrase binding domain causes a partial delocalization of CENPC *in vivo*. The data suggest that centromeric RNA helps to recruit CENPC to the inner kinetochore by altering its DNA binding characteristics.

## Author Summary

Here we address the issue of how genetic information is passed from one generation to the next without the involvement of specific DNA sequences. This type of inheritance is referred to as epigenetics. Centromeric sequences are highly variable and in many cases are not sufficient for centromere function. Rather, secondary features of the DNA, such as methylation or associated RNA molecules may serve to recruit key centromere binding proteins. Prior data from several species have established that single-stranded RNAs are surprisingly abundant on centromeric chromatin. Here we identified the DNA-binding domain of a key centromere binding protein in maize (CENPC) and showed that it requires single-stranded RNA to effectively bind DNA *in vitro*. When the DNA/RNA binding domain was deleted, the accuracy of CENPC targeting to centromeres was reduced but not abolished. The results bolster the view that centromere-bound RNA is one component of the epigenetic determination process that assures centromeres are stably inherited. In addition, our data suggest a general mechanism for how RNA can influence the binding of chromatin proteins to DNA.

## Introduction

Centromeres are important features of the genome that connect chromosomes to spindles. The connection occurs through a large multifunctional kinetochore complex that binds DNA, binds microtubules, and regulates the timing of anaphase [1]–[4]. Most plant and animal genomes contain diagnostic repeats that can be used to identify centromere boundaries, but unlike in fungi [5],[6], these sequences are not always necessary for organizing a functional kinetochore [7]–[9]. How kinetochores are accurately replicated is unknown and generally described as epigenetic–meant in the broadest sense that it is not easily classified as genetic.

Functional centromere domains are marked by a histone H3 variant known as Centromeric Histone H3 (CENH3) that has received intensive scrutiny as an important epigenetic identifier of centromeres [5]. In the absence of CENH3, all other kinetochore proteins fail to localize and chromosomes cannot move on the spindle [10]–[13]. CENH3 is assembled relatively late in the cell cycle, as late as anaphase-G1 [14]–[16], by specialized CENH3 nucleosome assembly factors such as Mis16 and Mis18 [17]. In addition several proteins that require CENH3 for localization also serve to target new CENH3 [18]–[20]. One such protein is Centromere protein C (CENPC), a DNA binding protein that has a key role in centromere recognition and maintenance [21]. Drosophila CENPC is required to target CENH3, but CENH3 is also required to target CENPC [20],[22]. These data suggest that kinetochore replication is a self reinforcing process whereby key inner kinetochore proteins such as CENPC work in concert with CENH3 to replicate the content and position of centromeres.

In species such as maize, the available data suggest that centromeric DNA does not function to recruit kinetochores until it is combined with specific epigenetic marks. Maize centromere repeats are under-methylated [23] and transcribed to produce stable RNAs that remain tightly bound to chromatin [24]. The centromeric RNAs are 40–200 nt in length, transcribed from both strands, and maintained in the single stranded state. It was proposed that RNA may serve as a structural template to help recruit kinetochore proteins [24]–[26]. A recent study revealed that human centromeres contain similar RNAs and established that RNAse treatment delocalizes CENPC from mature kinetochores [27]. More recent data show that suppression of transcription over a single LINE element in a human neocentromere impairs the formation of a kinetochore complex [28]. These data support the view that RNA facilitates or stabilizes the association of kinetochore proteins with centromeric DNA and implicate CENPC as a primary target of this activity.

CENPC is a highly divergent protein defined by a short 23 amino acids motif. Outside the defining motif lies the DNA binding region(s), which in animals appear to be distributed in several broad domains [29]–[31]. In plants, there is no evidence that CENPC binds to centromeric DNA beyond the presumption that CENPC is functionally conserved. In this regard we were encouraged by the sequence analysis of Talbert and colleagues [32] who found that a small region of CENPC has been repeatedly duplicated in the grasses. The authors suggested that the exon 9–12 duplicated region may bind to DNA. Here we use a combination of *in vitro* and *in vivo* studies to show that maize CENPC has both DNA-binding and RNA-binding capacities, that the DNA/RNA-binding domain is localized to the exon duplication region, and that the binding domain is required for efficient centromere localization. We further show that RNA directly facilitates the binding of CENPC to DNA *in vitro*, providing a biochemical mechanism for the involvement of RNA in centromere specification. We argue that CENPC and RNA are a part of the template that directs CENH3 to newly replicated centromere DNA.

## Results

### CENPC binds to DNA

**Figure 1:**
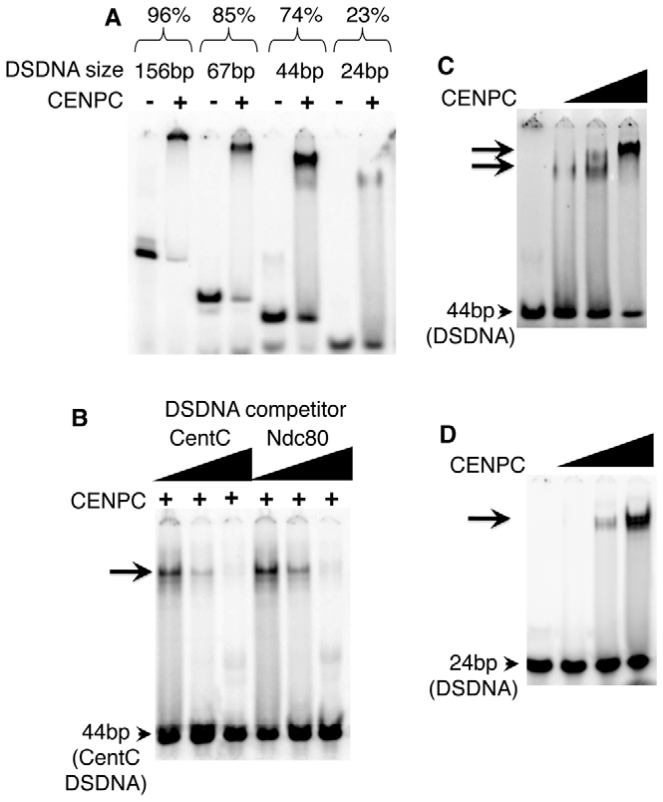
Maize CENPC binds to DNA. (A) Purified full CENPC and different sized CentC DSDNA binding substrates (indicated at top). The binding efficiency as % binding is shown (bound DNA/total). (B) Competition experiments. Lanes were loaded with the mixture of full CENPC protein, 44 bp CentC DSDNA, and either unlabeled 44 bp CentC or 44 bp Ndc80 DSDNA. (C) Increasing quantities of CENPC cause a supershift on a 44 bp substrate. (D) Increasing quantities of CENPC do not cause a supershift on the 24 bp substrate. The triangles represent the amount of protein or DNA added, and arrows indicate the shifted bands.

In animals, CENPC is a non-specific DNA binding protein *in vitro* [29]–[31]. As a first step towards understanding the biochemical properties of maize CENPC, we used a standard gel shift assay to test whether the full-length protein binds DNA. We used double stranded CentC DNA as the binding substrate. CentC is the primary tandem repeat in maize centromeres [33] and a suspected binding substrate for CENPC, although this interaction has not been shown directly. Bacterially expressed CENPC was mixed with ^33^P-labeled full-length CentC DNA (156 bp) and the products resolved by non-denaturing PAGE. Consistent with expectations, the data show that the mobility of CentC is shifted upwards in the presence of CENPC, indicating that DNA and protein are associated in a complex that slows migration on gels (Figure 1). As controls we used bovine serum albumin and maize NDC80, another kinetochore protein [34]. There was no gel shift with either control protein (data not shown).

CentC fragments of different lengths were incubated with CENPC to identify the optimal binding substrate. As shown in Figure 1A, CENPC complexes form with increasing efficiency as DNA length increases: 23.1% (shifted) with a 24 bp fragment, 73.7% with a 44 bp fragment, 85.3% with a 67 bp fragment, and 95.9% with a 156 bp fragment. Since the 67 bp and 156 bp fragments produced complexes that were too large to enter the polyacrylamide matrix, we opted for a 44 bp probe in subsequent assays.

The effects of CENPC concentration on DNA binding was investigated using 44 bp and 24 bp molecules. When the amount of CENPC was increased, a second, supershifted band was observed on the 44 bp template (Figure 1C), but not the smaller 24 bp DNA template (Figure 1D). These data suggest that larger DNA strands (44 bp) can accommodate two forms of CENPC-DNA complex. The stoichiometry could be skewed in either of two ways: Either there is more than one CENPC protein per DNA molecule on the supershifted band (e.g. two CENPC/one DNA), or each CENPC binds to more than one DNA (e.g. one CENPC/two DNA). The first option seems more likely since the shift occurs as more CENPC is added. Further, if CENPC could bind to a second (or third etc) DNA molecule at high concentrations, we would expect the same type of supershift with a 24 bp DNA fragment.

### DNA binding is not sequence-specific

Binding specificity can be determined by competition experiments in which unlabeled DNA (‘challenger’) is added as a competitor to a mix of protein and labeled (‘defender’) DNA. If the DNA-protein binding is sequence-specific, the defender DNA will not be competed away by the challenger DNA [35]. Here, three sequences were used as competitors. A repetitive knob repeat found on chromosome arms [36], a centromere repeat from sorghum [37], and a fragment of the single-copy *Ndc80* gene [34], were all efficient competitors for CENPC binding (*Ndc80* is shown in Figure 1B). In no case did CENPC appear to bind with higher affinity to CentC than other molecules. While it remains possible that CENPC has minor binding preferences *in vitro*, our results suggest that the differences (if any) cannot be reliably detected by the gel shift assay. These results reinforce the interpretation that CENPC targeting to centromeres is DNA sequence-independent.

### Maize CENPC binds both DNA and RNA at exons 9–12

**Figure 2:**
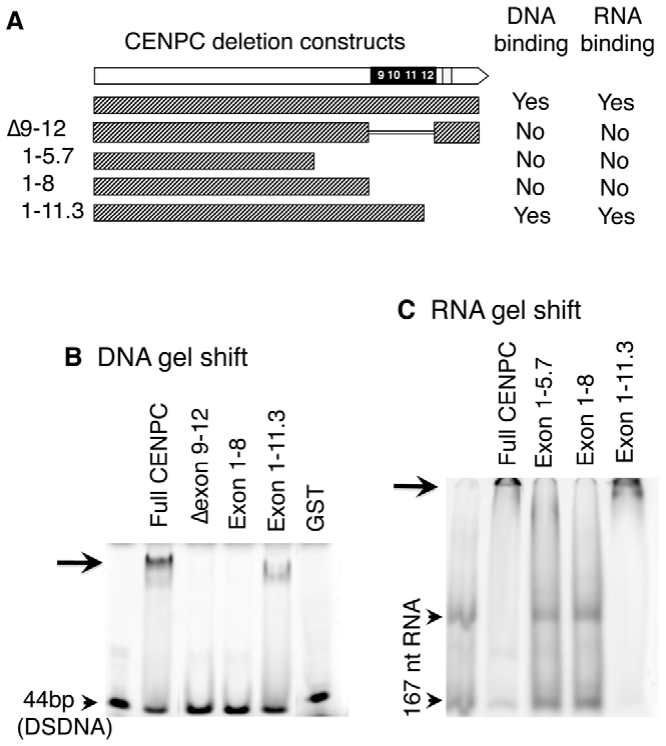
DNA and RNA binding localize to exon 9–12. (A) Schematic representation of CENPC constructs used. (B) DNA binding. Radiolabeled 44 bp DSDNA was incubated with equal amounts of the different CENPC fragments. A shifted band (arrow) was seen for full CENPC and Exon 1–11.3, but not for Δ Exon 9–12, Exon 1–5.7 or Exon 1–8. As a negative control, GST (Glutathione S-transferase) was tested and no band shift was seen. (C) RNA binding. Radiolabeled 167 nt RNA was incubated with equal amounts of different CENPC fragments. Free RNA appears as two bands as expected for a long RNA with double stranded character (arrowheads). A shifted band, which is too large to enter the gel matrix (but visible, see arrow), was seen for full CENPC and Exon 1–11.3, but not for Exon 1–5.7 or Exon 1–8.

To determine the DNA binding sites on maize CENPC empirically, fourteen subdomains of CENPC were tested for their capacity to shift DNA on gels (examples in Figure 2A–2C). The amount of protein required to confer a quantifiable shift was used as a measure of binding affinity. The data reveal that full-length CENPC has the highest DNA binding and that partial proteins bind DNA much less efficiently. By comparing the locations of the subdomains we inferred that the major DNA binding region maps to an area between exons 9 and 12 (Figure 2A). To confirm this interpretation, we prepared a construct that is identical to full length CENPC, but deleted for the entire 122 amino acid region containing exons 9–12 (Δexon 9–12). Gel shift results reveal that Δexon 9–12 has no detectable DNA binding activity (Figure 2B).

**Figure 3:**
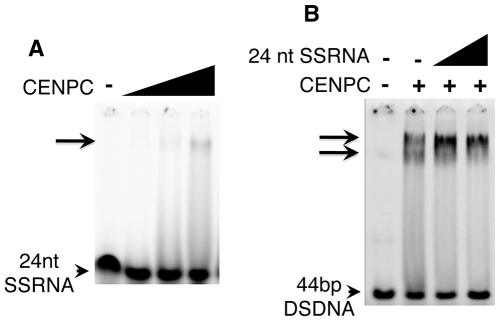
SSRNA causes a supershift of the CENPC/DNA complex. (A) 24 nt SSRNA binds weakly to CENPC. A faint shifted band is seen when CENPC is added at high concentrations. (B) Increasing amounts (triangles) of unlabeled 24 nt SSRNA cause the formation of a supershifted band similar to what is observed when excess CENPC protein is added (Figure 1C).

Since centromere/kinetochores are rich in RNA [24], CentC RNA was also used in gel shift assays. As shown in Figure 2C, CENPC is an RNA binding protein. Analysis of several constructs suggests that the RNA binding is conferred by the same exon 9–12 region that binds DNA. RNA transcribed from either strand of synthetic sequences containing a 134 bp sorghum centromeric repeat [37], the 156 bp CentC repeat, and the non-centromeric maize 180 bp knob repeat [36] were roughly equivalent in their affinity for CENPC. We also tested whether CENPC can bind small single stranded 24 nt RNAs homologous to CentC (SSRNA). The results show that CENPC does bind to the small RNA (Figure 3A), though with much lower efficiency than to longer SSRNA or to double-stranded DNA (Figure 2B and 2C).

### RNA promotes DNA binding by CENPC

The synthesized 24 nt SSRNA was used in competition assays with double stranded CentC DNA (Figure 3B). We found that CENPC DNA binding is not affected by adding excessive small RNA competitor (unlike DSDNA challengers). Instead, small RNA promotes the formation of a larger supershifted product (Figure 3B). The mobility of the RNA-supershifted band is similar or identical to the band observed when more CENPC is added (Figure 1C), which we argue represents two or more CENPC proteins on a single DNA substrate. CENPC without exon 9–12 (Δexon 9–12) was also tested for RNA-stabilized DNA binding and the results suggest that RNA has no effect on other domains (data not shown).

**Figure 4:**
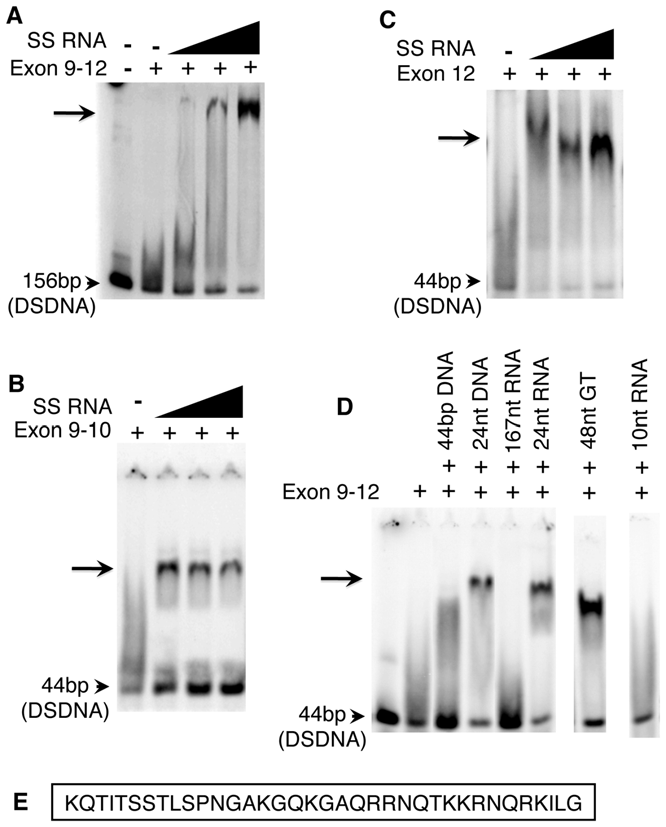
The purified Exon 9–12 domain requires RNA to bind DNA *in vitro*. (A) The DNA binding domain of CENPC alone does not bind to DNA, however a shifted band becomes evident as increasing amounts of SSRNA are added. The same is true for Exon 9–10 (B) and exon 12 alone (C). (D) Only single-stranded RNA or DNA stabilizes Exon 9–12 for DSDNA binding. Gel shift is observed in the presence of 24 nt SSDNA, 24 nt SSRNA, and 48 nt poly-GT (SSDNA). However, no shift is observed for 44 bp DSDNA, 167 nt RNA (with double stranded character) or 10 nt RNA. (E) Sequence of CENPC exon 12.

Remarkably, the DNA binding region alone does not bind DNA efficiently. However, when small RNA is added concurrently, a clear and strongly shifted band appears (Figure 4A). When a 4,000-fold molar excess of 24 nt SSRNA (relative to DNA) was added to the reaction, the effect was indistinguishable from the effect when small amounts were added. To further study this effect, we prepared an exon 9–10 peptide (Figure 4B) as well as a single exon 12 peptide (Figure 4C), and showed both of the smaller polypeptides also require SSRNA to bind DNA effectively. These data demonstrate that single stranded RNA does not compete with DNA binding but instead has a clear positive effect on DNA binding, perhaps by resolving a folding defect in the expressed protein.

### Other single-stranded nucleic acids stabilize the CENPC–DNA interaction

The majority of centromere-associated CentC and CRM transcripts are single-stranded and larger than 40 nt [24]. To test whether RNAs of this type are effective CENPC stabilizers, we tested a variety of single- and double-stranded nucleic acids in the gel-shift assay. We found that both 24 nt SSRNA and SSDNA increase the association of CENPC and DNA (Figure 4D), suggesting that single-strandedness is the key stabilizing feature. However, very short single-stranded RNA (10 nt) had no effect on the CENPC-DNA interaction (Figure 4D). These data suggest that that the stabilization event required oligomers with a minimum length, and that multiple very small RNAs cannot compensate for a proportionally longer RNA.

Tests of longer molecules are confounded by the fact that single-stranded nucleic acids tend to form hairpin secondary structures based on partial homology. Long 167 nt transcripts containing CentC and double stranded small molecules (Figure 4D) did not stabilize the CENPC-DNA complex but instead competed in the binding reaction. Therefore, we used a long (48 nt) DNA polydinucleotide with a repeating GTGT motif that cannot form a double-stranded state. The GTGT polynucleotide stabilized the CENPC-DNA binding reaction efficiently, similar to small RNA (Figure 4D). Thus, long single-stranded nucleic acids similar to those present *in vivo* [24] are effective stabilizers of CENPC binding *in vitro*.

We performed the same tests on the well-studied HIV Integrase DNA-binding domain (IntBD) [38], which binds DNA non-specifically similar to CENPC. IntBD is also similar in size to the DNA binding modules in maize CENPC (51 amino acids as compared to 61 amino acids for either exons 9–10 or exons 11–12). The IntBD region was synthesized *in vitro* and used in DNA gel shift assays. The data show that IntBD binds strongly to DNA without the need for RNA (Figure S1). When RNA is added to the IntBD-DNA mixture, there was no observable effect. These data suggest that stabilization by RNA is a unique feature of the CENPC DNA-binding domain.

### The exons 9–12 domain is necessary *in vivo* for accurate CENPC targeting

**Figure 5:**
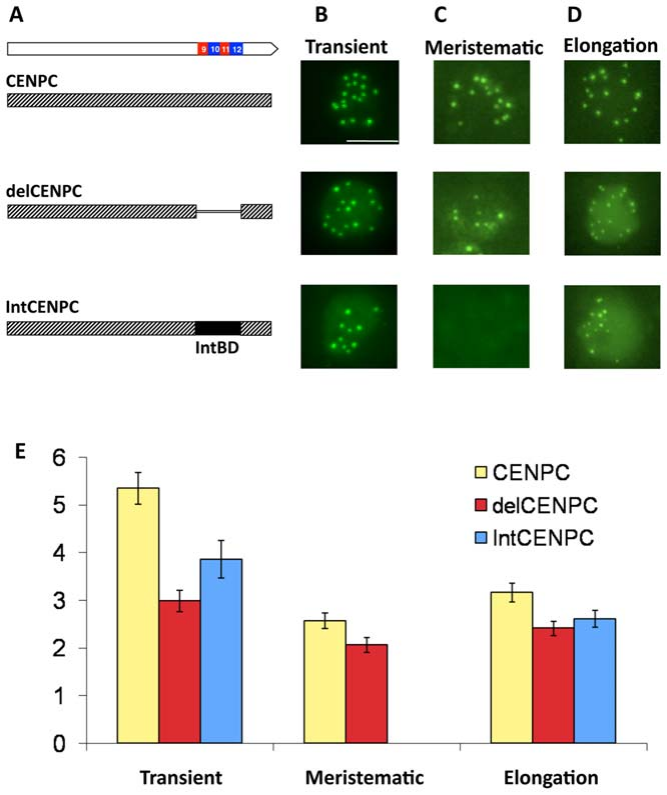
Removal or replacement of Exon 9–12 delocalizes CENPC *in vivo*. (A) Schematic representation of the YFP–CENPC constructs used in maize transformation. (B) Transient expression of YFP–CENPC in cultured cells. The green YFP spots represent kinetochore localization, as determined from fixed cells (Figure 6). (C) Fluorescence in root tips of stably transformed plants. (D) Fluorescence in the elongation zone of stably transformed plants. Images are projections showing all YFP signal (green) from single nuclei. (E) Quantification of the data in (B–D). The Y axis represents the ratio of YFP signal in kinetochores to YFP signal in the nucleoplasm. There are significant differences between full CENPC and delCENPC and IntCENPC in all three types of tissue (P<0.05, bars show SEM).

We tested the function of the CENPC binding domain *in vivo* using two assays, transient and stable. Transient transformation was used to provide a large sample size while stable transformation was used for more detailed assessments of tissue specificity and heritability. Transient assays were conducted by biolistic transformation of embryogenic callus surface cells. Three constructs were tested: the full length CENPC gene, a gene with exon 9–12 deleted (delCENPC), and a construct with the natural exon 9–12 replaced with HIV IntBD (IntCENPC, Figure 5A). The genes were constitutively expressed under control of the 35S promoter and CENPC was tagged by YFP at the N-terminus (our preliminary studies revealed that YFP at the CENPC C-terminus impairs kinetochore localization). Assays from transient transformation revealed that deletion of the DNA/RNA binding domain (delCENPC) reduced centromere localization to 56% (n = 39 cells) while substitution of exon 9–12 with IntBD decreased centromere localization to 72% (n = 32 cells; Figure 5A, 5B, and 5E). These data show that exon 9–12 is necessary for efficient centromere targeting in tissue-cultured interphase cells.

**Figure 6:**
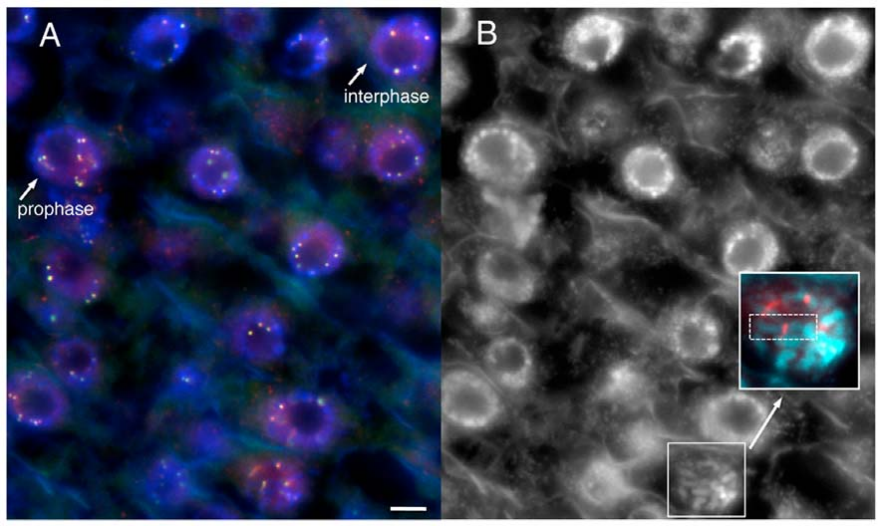
YFP–CENPC localizes to kinetochores. (A) A cryostat section from a root tip, showing cells in various stages of the cell cycle. YFP–CENPC is labeled by anti–YFP antisera (red), while YFP itself is shown in green. The red and green signals overlay to produce a yellow color. DNA (DAPI) is shown in blue. Cells in interphase and prophase are noted and differentiated by chromatin staining. Kinetochores on prophase chromosomes are noted with arrows to show the paired spots on replicated chromatids. (B) A black and white version of the DNA stain in (A). An early prophase cell is enlarged in the panel to highlight a single chromosome, with anti–YFP staining (red) lying in the primary constriction. Each kinetochore is noted with an arrow. Bar = 5 µm.

The same constructs were then introduced into whole plants by *Agrobacterium*-mediated transformation [39]. Fixed cells were used to confirm that the transformed YFP–CENPC protein localizes to kinetochores at all stages of the cell cycle (Figure 6). The data show that the number of YFP-positive spots in interphase is twenty or fewer (usually 15–20; some kinetochores stick together), and that in prophase the kinetochore spots are paired and limited to primary constrictions, consistent with prior observations [34]. Live-cell assays were then used to quantify the efficiency of localization (by comparing kinetochore to nucleoplasm fluorescence). Two cell types were assayed: root tips, which are rich in actively dividing cells, and elongation zone cells, which are older and undergo few divisions. Deletion of the binding domain (delCENPC) reduced centromere localization to ∼80% of full CENPC in both tissue types (Figure 5C–5E, and Figure S2). In contrast, replacement of exon 9–12 with IntBD abolished kinetochore localization in root tips (Figure 5C, Figure S2). Nevertheless kinetochore localization of IntCENPC recovered in elongation zones and accumulated to ∼80% of the full-length CENPC control (similar to delCENPC; Figure 5D and 5E, and Figure S2). These data suggest that DNA/RNA binding increases the affinity of CENPC for intact centromeres, and that the DNA/RNA binding region is most important during cell division.

### Native CentC transcripts are predominantly 75 nt and transcribed from one strand

**Figure 7:**
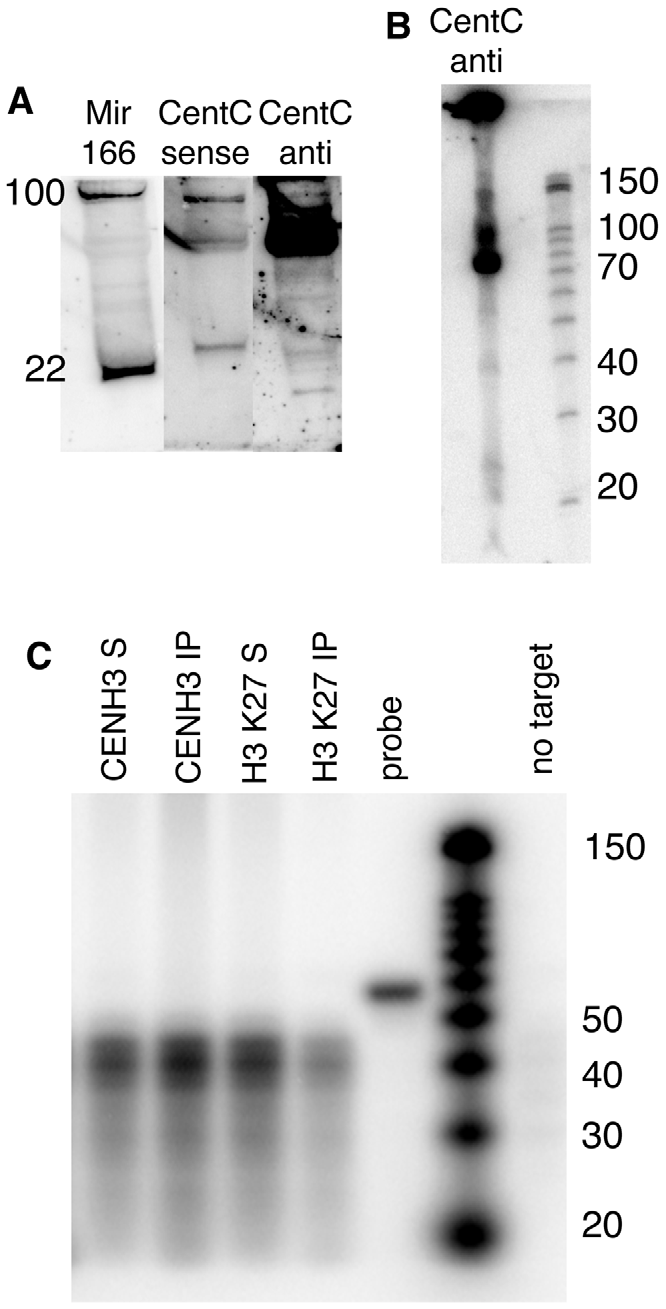
Chromatin-associated CentC transcripts are predominantly 75 nt and do not include siRNAs. (A) Northern blot of total maize RNA. DNase-treated total RNA (enriched for ≤200 bp) was separated by PAGE and blotted to a membrane. Radiolabeled RNA probes specific to microRNA166 (miR166) [55] and CentC forward (GenBank AY530283.1) and reverse strands were hybridized in succession. The mature form of miR166 is 22 nt while its precursors are ∼100 nt and seen near the top of the gel. Molecular weights were estimated by ethidium bromide staining of 22 and 28 nt RNA oligonucleotides (not shown). (B) Higher resolution RNA blot showing the size classes of CentC forward transcripts. Molecular markers are shown on the right. (C) Analysis of RNA associated with CENH3 and H3-containing chromatin. RNA recovered from ChIP experiments (3.34 µg) was subjected to RNase protection (RPA) using the CentC forward probe (52 nt). The upper resolution of detectable sizes is 44 nt (8 nt less than the probe size). The fraction associated with immune complex is labeled ‘IP’ and the non-associated fraction is labeled ‘S’. Yeast RNA was used to demonstrate complete digestion in the absence of target DNA, and is labeled ‘no target’.

In order to better understand the nature of centromeric transcripts *in vivo*, we subjected total maize RNA to a careful analysis. The intent of these experiments was to identify the full suite of centromeric RNAs in maize. Our prior work had focused only on those RNAs associated with chromatin, and did not reveal an siRNA-sized class [24]. Total cellular RNA was assayed by a standard northern protocol (Fure 7A and 7B). These data show that the majority of CentC RNAs are discretely-sized, but much longer than micro or siRNAs (compare to miR166 at 22 nt; Figure 7A). At higher resolution it is clear that the major form is 75 nt. Most of the transcripts originated from the ‘forward’ strand of CentC (as defined by AY530283.1; Figure 7A), although both strands are abundant in CENH3-associated chromatin [24]. Other longer transcripts(s) are also present, as well as a 40 nt band seen previously [24], and the predicted siRNA-sized bands (Figure 7B). Centromeric RNAs of similar size have also been observed in other species [40]–[43].

### Native CentC transcripts are not exclusively present at maize centromere cores

Prior research established that long single stranded transcripts are associated with maize centromeric chromatin [24], but did not address the question of whether similar transcripts can be associated with pericentromeric (or other) regions. Therefore we carried out ChIP experiments with antisera to H3K27me1, an abundant form of histone H3 in pericentromeric areas [44], and compared it to CENH3 ChIP. An RNase protection assay (RPA) was used to gain a sensitive measurement of the CentC RNA associated with ChIP samples (Figure 7C). The data reveal that long CentC RNAs are associated with CENH3 chromatin, while siRNAs are not, despite the fact that siRNAs can be observed in total RNA preparations (Figure 7A and 7B). We also observed detectable (though lesser) quantities of CentC RNAs associated with H3K27me1-containing nucleosomes. This result was confirmed in a second experiment using a real time PCR as the assay (see Materials and Methods). Therefore we do not suppose that kinetochores are unique in retaining RNA on chromatin, but rather that centromeric RNAs are more abundant within kinetochore domains.

## Discussion

This study was designed to identify the DNA binding characteristics of CENPC, which in plants is presumed to be the primary protein that binds to the surface of centromeric DNA. Our intent was to test the idea that CENPC is a principle factor in conferring heritability to centromeres. Prior data indicated that single-stranded RNA is abundant in maize centromeric nucleosome purifications [24], that maize CENPC contains an adaptively evolving exon duplication domain [32], that in animals single-stranded RNA is required to maintain CENPC at kinetochores [27], and that transcription of a LINE retroelement is required for kinetochore maintenance over a human neocentromere [28]. Here we provide data that suggest an underlying mechanism for these observations–that centromeric RNA provides a means for CENPC to effectively bind DNA.

In animals the DNA binding region of CENPC is poorly defined and shows no homology to CENPC homologs in non-mammalian species. The lack of homology outside of a 23 aa acid region (of unknown function) has been cast as evidence that the protein is under selection to adapt to DNA sequence change [32]. The argument is perhaps strongest in the grasses, where a small exon 9–12 region has been repeatedly duplicated as if under diversifying selection [32],[45]. Maize CENPC exon 9–12 is rich in arginine and lysine similar to other DNA binding regions [46],[47]. Our study was initiated in part to test the hypothesis that exon 9–12 is indeed the primary DNA binding region.

A comprehensive truncation/deletion analysis confirmed that nearly all DNA and RNA binding activity in maize CENPC lies within the exon 9–12 domain (Figure 2A–2C). The binding reaction lacks sequence specificity, to the extent that any double stranded nucleic acid competes with the native CentC repeat in gel shift assays. Single-stranded molecules, in contrast, do not compete but instead strongly promote DNA binding. The addition of RNA causes full CENPC to bind as a supershifted product that we associate with multiple CENPC proteins per DNA substrate (Figure 3B). The RNA effect is much more dramatic with the purified DNA binding module alone, which cannot bind DNA efficiently unless single-stranded nucleic acids are present (Figure 4A). These data may suggest that the DNA binding module is naturally unstructured [48], perhaps in a way that blocks the protein from folding or dimerizing properly.

Further studies revealed that the stabilizing molecules must be single-stranded and larger than 10 nt (Figure 4D), that excessive SSRNA does not compete with a DNA-CENPC complex, and that RNA stabilizes a single 36 amino acid-binding module that is probably too small to accommodate the binding of both DNA and RNA (Figure 4C). These data suggest that RNA binds transiently to CENPC and alters CENPC structure to facilitate DNA binding, similar to the function of a protein chaperone. RNA-stabilized DNA binding may be a unique feature of CENPC, since our assays show that another DNA-binding domain (HIV IntDB) lacks this property (Figure S1).

Our *in vitro* observations correspond well to the observation that maize centromeric chromatin is rich in 40 to 200 nt single-stranded RNAs [24]. We have shown here (Figure 7C) and previously [24] that long SSRNAs are preferentially associated with CENH3 domains. The most abundant forms are discretely sized at 75 nt and 40 nt (Figure 7A and 7B), while siRNAs, which are detectable in total RNA extracts (Figure 7A and 7B), are not associated with CENH3 chromatin (Figure 7C). Notably, several groups have found centromeric RNAs of similar size, suggesting the possibility of a processing system distinct to centromeres [24], [41]–[43],[49].

Prior data from human cells strongly suggest that RNA-facilitated DNA binding is a conserved feature of CENPC. Human CENPC contains a nucleolus-localizing sequence (NoLS) that is essential for SSRNA binding [27], and the same RNA binding region is necessary for CENPC centromere localization [27]. The authors argue that human CENPC assembles as an RNA-containing pre-kinetochore complex in the nucleolus before being incorporated into centromeres. Although we do not see CENPC nucleolar localization in maize, it is likely that RNA serves to stabilize a CENPC protein complex in both human and maize. In a second report, a human neocentromere (mardel 10) was shown to contain a single actively transcribing LINE retrotransposon at the core of the CENH3 domain. The authors showed that LINE transcripts are incorporated into CENH3 chromatin and necessary for kinetochore replication in dividing cells [28].

### A model for RNA facilitated exon duplication binding in centromeric assembly of CENPC

**Figure 8:**
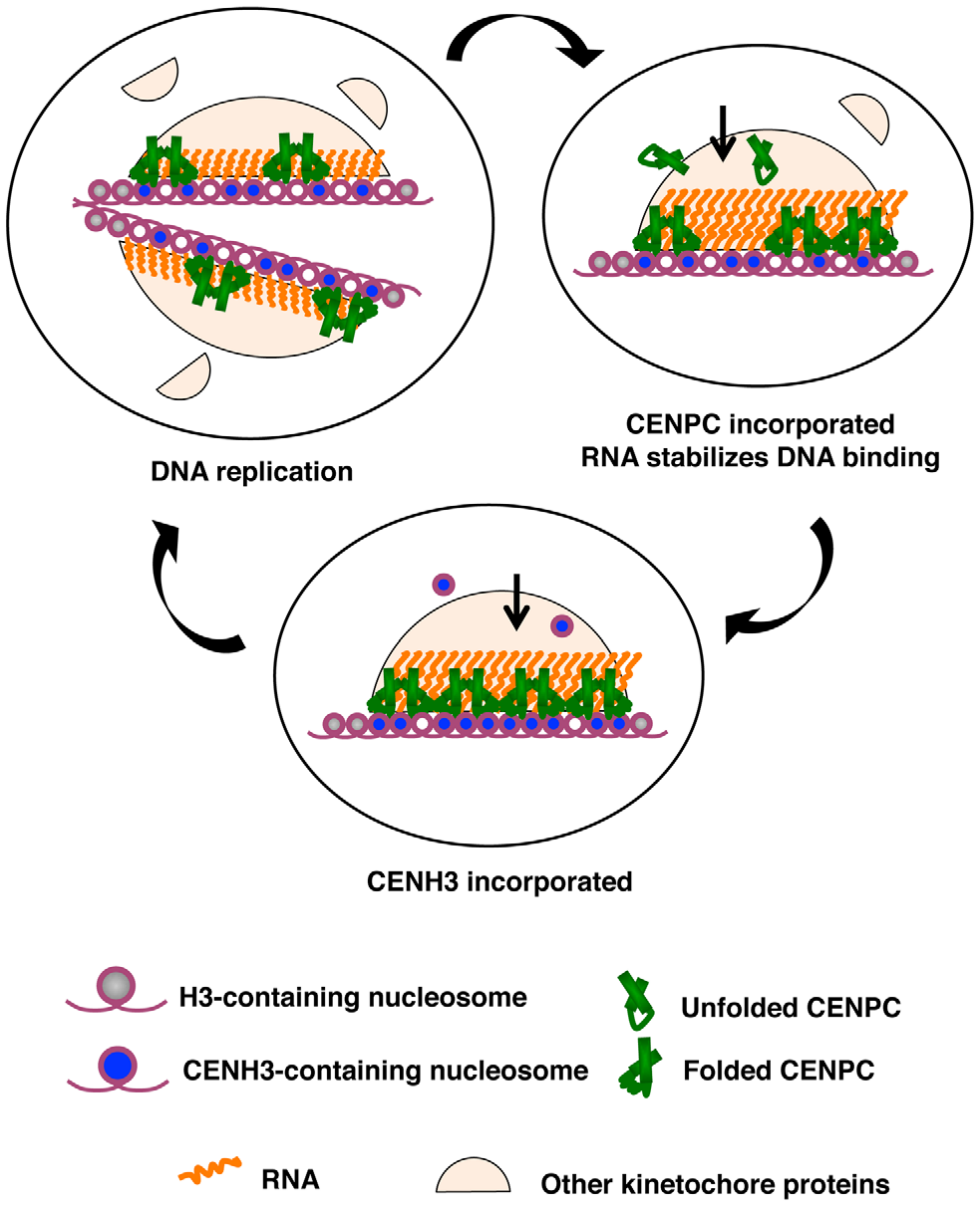
A model for how RNA facilitates the CENPC-DNA interaction. Three stages of kinetochore replication are shown in a cycle that broadly represents the cell cycle. DNA replication splits kinetochores and distributes the resident kinetochore proteins to sister chromatids. CENPC recruitment is continuous. Protein–protein contacts bring CENPC to the kinetochore while resident RNA stabilizes CENPC for DNA binding. CENH3 assembly is a discrete event that occurs after S phase, as early as G2 [14] or as late as anaphase or G1 [15],[16]. CENPC and CENH3 assembly are separated in time such that one can guide the other.

Our observation that the delCENPC and IntCENPC constructs, which lack the natural DNA binding domain of maize CENPC, localize to kinetochores with 80% efficiency (Figure 5) suggests that initial targeting of CENPC occurs independently of DNA binding. From these data we infer that the role of the DNA binding domain is to reinforce and/or stabilize accurate localization once it occurs. The fact that single stranded RNA has a strong positive effect on DNA binding suggests that centromeric RNA serves as an epigenetic mark that mediates this final and most stable state of assembly. Therefore we propose that CENPC is first recruited to kinetochores by protein-protein contacts and then converted to a functional DNA binding protein by centromeric RNA (Figure 8). We emphasize that our interpretation relies heavily on *in vitro* experiments, and that our *in vivo* data are complicated by the presence of wild type CENPC in the transformed lines, which may have influenced the recruitment of introduced YFP-tagged proteins. Although our data show that DNA binding has a role in maize CENPC function, our experiments cannot be used to quantify how important DNA binding is; nor do our data establish with certainty that RNA facilitates DNA binding *in vivo*. Nevertheless our data provide the first plausible mechanistic explanation for a previously unexplained phenomenon.

The role of RNA in CENPC function may be similar in principle to the well-understood roX1 and roX2 RNAs of Drosophila, which function to up-regulate genes on the male X chromosome [50]. roX RNAs bind to a small complex of MSL (male-specific lethal) proteins and change their binding specificity so that they interact with many sites along the X chromosome [50]. Without roX RNAs, the MSL complex loses its specificity and dosage compensation is lost. Similarly, most of the centromeric RNA in maize probably functions in trans; that is, it is unlikely that all RNA associated with centromeric DNA is encoded by the DNA directly beneath it. However it is also very probable that each centromere has the capacity to transcribe its own large population of RNA. The transcriptionally competent state of each centromere [23] can provide a renewable, effective cis-acting source of RNA for stabilizing CENPC at the inner kinetochore.

In the larger view, the mode of centromere replication may revolve around that fact that two proteins can serve as place markers, CENH3 and CENPC, and that they have different temporal patterns of incorporation. CENH3 is assembled in a defined period (G2 in plants, G1 in animals), while CENPC is incorporated throughout the cell cycle [16]. If, as in Drosophila, the two proteins can target each other to the kinetochore [20],[22], then at least one of the place markers will be present on DNA at all times (Figure 8). While CENH3 appears to be a defining feature in all eukaryotes, any number of other kinetochore proteins could serve as the second marker and target CENH3 or its assembly factors. CENPC is currently the best candidate in plants, but it is conceivable that other known or unknown kinetochore proteins, such as the functional equivalent of mammalian CENP-I or CENP-H may have similar roles [16],[18],[19].

## Materials and Methods

### Preparation of recombinant CENPC protein and its variants

Full CENPC (1–701 aa), Δexon 9–12 (1–502 aa+625–701 aa), exon1–5.7 (1–399 aa), exon1–8 (1–502 aa), and exon1–11.3 (1–601 aa) were amplified from the CIMMH01 plasmid (GenBank AF129857) [51]. To generate Δexon 9–12, a 1∼1506 bp fragment at the 5′-end and an 1873∼2106 bp fragment at the 3′-end of the maize *Cenpc* gene were first amplified separately. The two amplicons were then joined together by overlapping ends in a secondary PCR. The same strategy was used to generate IntCENPC except that an amplified DNA-binding domain of HIV integrase (GenBank AAC83550, amino acids 220–270) [38] was added as a third template for secondary PCR.

All constructs were inserted into the pET28a vector (Novagen) and transformed into Rosetta Blue (DE3) competent cells (Novagen). Recombinant CENPC subdomains were expressed as histidine-fused proteins and purified according to the manufacturers protocols. The expressed proteins were verified by size using His-tag staining (Invitrogen) and western blotting. The peptide for exon 12 of maize CENPC was synthesized by Sigma-Genosys and the peptides for CENPC exon 9&10 and the DNA-binding domain of HIV integrase were synthesized by Abgent.

### Preparation of DNA probes and competitors

A 156 bp CentC monomer identical to GenBank AF078922 was synthesized by annealing two long primers and cloned into the pCR4 vector (Invitrogen). The 67 bp CentC probe was generated using primers GGTTCCGGTGGCAAAAACTCGTGC and GCACGTCACCCATTCTGAAAACGG. Shorter single-stranded DNA sequences were synthesized as oligonucleotides, and if needed, annealed with a complementary oligonucleotide to form duplexes. These were the 44 bp CentC probe AATGGGTGACGTGCGGCAACGAAATTGCGCGAAACCACCCCAAA, the 24 bp CentC probes CCGTTTTCAGAATGGGTGACGTGC, and a 44 bp fragment of the maize Ndc80 cDNA. For gel shift assays, DNA was end-labeled with ^33^P-ATP using T4 polynucleotide kinase (Invitrogen).

### Preparation of RNA probes and competitors

CentC RNA was synthesized from a construct containing the SP6 promoter upstream of a sequence identical to GenBank AF078922. The 167 nt RNA was transcribed *in vitro* using Sp6 RNA polymerase and a Riboprobe kit (Promega). To label RNA, ^33^P-labeled UTP was added to the reaction. The 24 nt RNA rCrCrGrUrUrUrUrCrArGrArArUrGrGrGrUrGrArCrGrUrGrC and 10 nt RNA rCrCrGrUrUrUrUrCrArG molecules were synthesized by Integrated DNA Technologies and end-labeled with^ 33^P-ATP using T4 polynucleotide kinase (Invitrogen).

### Electrophoretic mobility shift assays

Radiolabelled DNA or RNA probes were incubated with CENPC or CENPC derivatives on ice for 20 min in a 20 µl solution of 10 mM Tris (pH 7.5), 50 mM NaCl, 0.5 mM dithiothreitol, 0.5 mM EDTA, 1 mM MgCl2 and 4% glycerol. For competition experiments, unlabeled DNA of different sequence was added in excess. The reaction mixtures were separated on 5% polyacrylamide gels and detected using a PhosphorImager.

### Generation of YFP–tagged CENPC constructs

CENPC, delCENPC, and intCENPC sequences were cloned into the pENTR/D-TOPO vector (Invitrogen). These were then recombined into the pEarleyGate 104 vector [52] using LR clonase (Invitrogen) to form N-terminal YFP fusions. Recombinant plasmids were transformed into Agrobacterium strain C58C1 for maize transformation.

### Plant transformation and analysis of YFP expression *in vivo*

Agrobacterium-mediated transformation of maize (hybrid line HiII) was performed by the Plant Transformation Facility at Iowa State University [39]. Transgenic plantlets were grown to maturity in the UGA Plant Biology greenhouse and crossed to inbred B73. Progeny were grown at 30°C and root tips observed *in vivo*.

The YFP–CENPC, YFP-delCENPC, and YFP-IntCENPC plasmids were also used for particle bombardment of maize HiII callus. Transient transformation was performed with plasmid DNA-coated gold particles using a PDS1000 system (Bio-Rad). The bombarded callus was cultured in dark for 18 h prior to observation.

### Immunolocalization

Root tips of transgenic plantlets were fixed in PHEMS buffer as previously described [53] and sections 10 µm in thickness were prepared on a cryostat (−20°C). Tissue sections transferred on polylysine slides were incubated with rabbit anti-YFP antibodies (1∶50) and then rhodamine-conjugated goat anti-rabbit antibodies (1∶100; Jackson Immunoresearch, West Grove, PA). The DNA was stained with 0.1 ug/ml 4,6-diamidino-2-phenylindole (DAPI).

Localization data were captured as 3D data sets by an Intelligent Imaging Innovations (Denver) Everest Digital Microscope Workstation and further analyzed by SlideBook 4.0 (Intelligent Imaging Innovations) and SoftWoRx (Applied Precision, Issaquah WA) software packages.

### Image capture, processing, and intensity analysis

Three-dimensional image sets of YFP localization were acquired using an Intelligent Imaging Innovations (Denver, CO, USA) Everest Digital Microscope Workstation. For the transient expression assays of YFP–CENPC, YFP-delCENPC and YFP-intCENPC, 60 cells, 39 cells, and 32 cells were sampled respectively. For the stable transgenic lines, all levels of assay were performed in triplicate. For each construct, progeny from three different transformants were analyzed. For each transformant, three different progeny were assayed. For each progeny, three meristematic areas and three elongation areas, each containing 20∼50 cells per area were assayed.

Image analysis was carried out using SlideBook 4.0 software. Maximum projection was obtained from each 3D stack and used for signal measurement. For each cell, kinetochores and nucleoplasm were masked and the sum intensities and volumes recorded. Background, calculated from four random areas, was subtracted from the intensity data. Kinetochore localization was calculated as the mean intensity of kinetochore staining divided by the mean intensity of nucleoplasm staining.

### Native centromere RNA analysis

Total RNA from young maize ears was isolated and enriched for RNA smaller than 200 nt using the mirVana miRNA Isolation Kit (Ambion), then treated with the TURBO DNA-free kit (Ambion). The quantity of RNA recovered was calculated using a NanoDrop (ThermoScientific). Samples were separated on 12% or 15% TRIS-UREA polyacrylamide gels and blotted to BrightStar-Plus charged membranes (Ambion) using 0.5X TBE in a semidry blotter. Radiolabeled probes were generated using the *mir*Vana miRNA Probe Construction Kit from Ambion, a T7 RNA polymerase based procedure that incorporates 32P-rCTPs internally at every cytosine. Template sequences were as follows, where lower case letters are T7 primer regions: CentC ‘forward’ TTTGGGGTGGTTTCGCGCAATTTCGTTGCCGCACGTCACCCATTcctgtctc, CentC ‘reverse’ AATGGGTGACGTGCGGCAACGAAATTGCGCGAAACCACCCCAAAcctgtctc, and mir166 TCGGACCAGGCTTCATTCCCCcctgtctc. Full-length probes were gel purified and measured by scintillation.

Northern blot hybridization was conducted using ULTRAhyb buffer (Ambion). Prehybridization was at 68° C for ≥1 hour, and hybridization was at 42° C for ≥12 hours, using a probe concentration of 10^6^ cpm/mL. Washes were at 42° C in 2X SSC and 0.1% SDS for 15 minutes. Similar results were obtained with additional forward and reverse probes that targeted other regions of CentC.

Chromatin immunoprecipitation was carried out as previously described [24]. H3K27me1 antibodies (Upstate 07-448) were used at a dilution of 1∶250. RNase protection assays on DNase treated samples were conducted using the RPA III kit (Ambion) with the CentC ‘forward’ probe. A PhosphorImager and ImageQuant software (Amersham Biosciences) were used to capture and quantify images.

Quantitative RTPCR was performed by standard methods. DNase I treated ChIP samples were reverse-transcribed using random hexamers and Superscript III enzyme (Invitrogen). Each sample was subsequently assayed using primer CentC-F1 GAAATGGTTCCGGTGGCAA and CentC-R1 TGGTTTCGCGCAATTTCGTT, or Zm5S-F1 GATGCGATCATACCAGCACTA and Zm5S-R1 GAATGCAACACGAGGACTT (to 5S ribosomal RNA). Relative fold enrichment (RFE) was calculated by the 2^−ΔΔCt^ method [54] using the 5S ribosomal RNA sequence as a control. Reactions were averaged from triplicate wells and normalized to controls lacking reverse transcriptase.

## Supporting Information

Figure S1 RNA does not influence the binding of purified HIV Integrase DNA binding domain to DNA. (A) Radiolabeled 44 bp DNA was incubated with increasing amounts of IntBD (HIV Integrase DNA binding domain) to reveal the shifted product. (B) Added SSRNA has no effect on IntBD.
(0.20 MB TIF)

Figure S2 Large-scale view of YFP expression in stably transformed plants. Panels (A–C) show root tips (dividing cells), while panels (D–F) show elongation zones (mature cells). The three constructs tested are indicated in the center. Removal of the exon 9–12 DNA binding domain (delCENPC) causes a 20% reduction in kinetochore localization. Replacement of exons 9–12 with HIV Integrase BD abolishes kinetochore localization in root tips. However, in elongation zone cells, IntCENPC localizes to kinetochore at roughly the same levels (20% reduction) as delCENPC. Bars = 10 µm.
(2.02 MB TIF)
