# Supplementary figures and images for: DNA Binding of Centromere Protein C (CENPC) Is Stabilized by Single-Stranded RNA
Source: PLoS Genet. 2010 Feb 5;6(2):e1000835. doi: 10.1371/journal.pgen.1000835 (PMC2816676; doi:10.1371/journal.pgen.1000835)

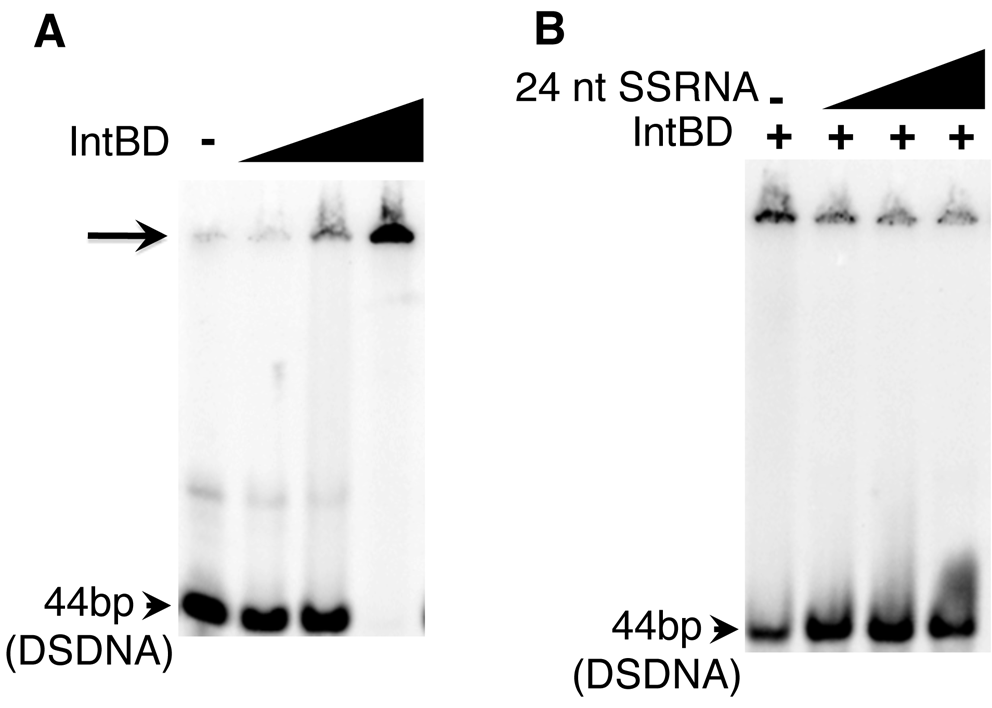

Supplement: Figure S1 — RNA does not influence the binding of purified HIV Integrase DNA binding domain to DNA. (A) Radiolabeled 44 bp DNA was incubated with increasing amounts of IntBD (HIV Integrase DNA binding domain) to reveal the shifted product. (B) Added SSRNA has no effect on IntBD. (0.20 MB TIF) [file pgen.1000835.s001.tif]

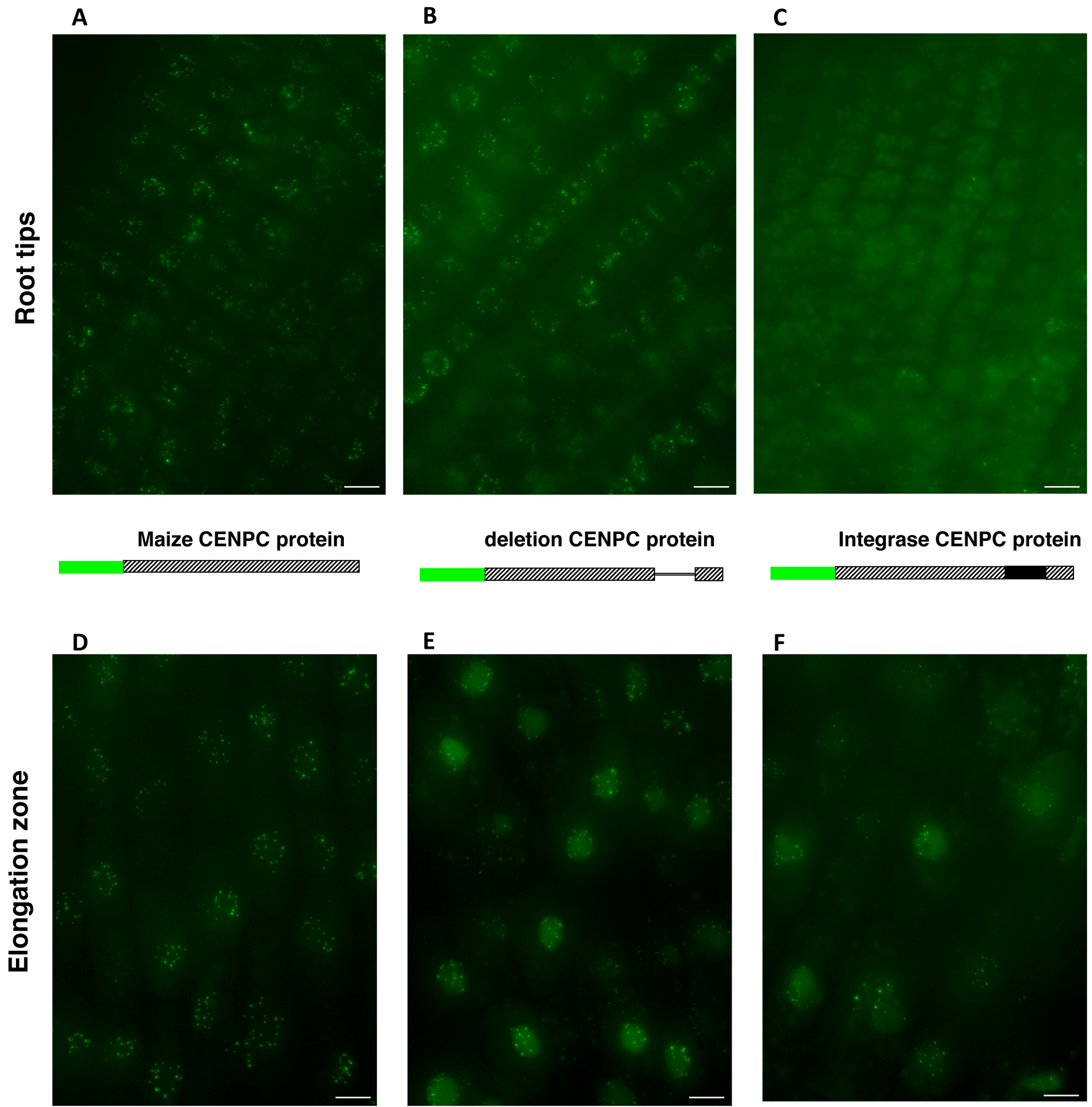

Supplement: Figure S2 — Large-scale view of YFP expression in stably transformed plants. Panels (A–C) show root tips (dividing cells), while panels (D–F) show elongation zones (mature cells). The three constructs tested are indicated in the center. Removal of the exon 9–12 DNA binding domain (delCENPC) causes a 20% reduction in kinetochore localization. Replacement of exons 9–12 with HIV Integrase BD abolishes kinetochore localization in root tips. However, in elongation zone cells, IntCENPC localizes to kinetochore at roughly the same levels (20% reduction) as delCENPC. Bars = 10 µm. (2.02 MB TIF) [file pgen.1000835.s002.tif]
